# Effect of Methylprednisolone on Ischemic Brain Edema After Temporary Occlusion of the Middle Cerebral Artery in Rats

**DOI:** 10.33549/physiolres.935600

**Published:** 2025-10-01

**Authors:** Petr KOZLER, Vít HERYNEK, Jaroslav POKORNÝ

**Affiliations:** 1 Institute of Physiology, First Faculty of Medicine, Charles University, Prague, Czech Republic; 2 Center for Advanced Preclinical Imaging, First Faculty of Medicine, Charles University, Prague, Czech Republic

**Keywords:** Temporary occlusion of the middle cerebral artery, Brain water content, T2 relaxation, Apparent Diffusion Coefficient, Methylprednisolone

## Abstract

We studied the effect of Methylpredisolone (MP) on ischemic brain edema after temporary occlusion of the middle cerebral artery for 90 min (MCAo90). We verified the presence of ischemic edema by determining the brain water content (BWC) by measuring dry/wet weight and by examining MRI – T2-weighted imaging, T2 relaxation times and apparent diffusion coefficient (ADC). In another group, animals were administered MP intraperitoneally 30 min after MCAo90, followed by 24 h reperfusion (MCAoMP). Edema changes were documented by the same MRI examinations. A statistically significant increase in BWC was found between the post-MCAo90 group of animals and the intact animals, demonstrating the presence of edema in the former group. A statistically significant increase in ADC was observed in the MCAo group, indicating the presence of vasogenic edema. A statistically significant difference was demonstrated between the MCAo and MCAoMP groups, with no statistically significant difference between the CG and MCAoMP groups, demonstrating a reduction in ischemic brain swelling after MP administration. The main effect of MP on ischemic brain edema is attributed to its antioxidant capacity. It can be assumed that this capacity of MP, with its complex impact on cellular metabolism, affects the movement of water in the brain and reduces ischemic brain edema.

## Introduction

It is a well-known fact that stroke is one of the most serious diseases ever. Annually, 15 million people worldwide suffer a stroke, of these, 5 million die and another 5 million are left permanently disabled [1]. There is also general agreement that ischemic edema is the cause of high morbidity and mortality in ischemic strokes [2]. The search for a successful treatment for focal cerebral ischemia is a continuous and long-term process that holds much promise in the field of experimental medicine, but has no obvious significance for the fate of patients [3].

It is interesting how much of a contradiction there is in the interest in studying this issue. This is well demonstrated by the search results in the PubMed database (30 January 2025). First, we entered the very general terms “ischemic stroke” (114,537 citations) and “ischemic brain edema“ (7,788 citations), giving a ratio of 14.5:1. We then narrowed the search to experimental models – “middle cerebral artery occlusion AND rat” (12,213 citations) and “ middle cerebral artery occlusion AND ischemic edema AND rat” (1,406 citations), corresponding to a ratio of 8.5:1. So the preference of study interests is obvious. The issue is comprehensively described in their current review by Simard *et al.* when they mention:

“...we highlight the often-neglected concept that brain edema and brain swelling are not simply secondary, correlative phenomena of stroke but distinct pathological entities with unique molecular and cellular mechanisms that are worthy of direct targeting” [3].

In light of the above, it seems defensible to test agents that reduce ischemic edema without affecting the pathophysiology of focal cerebral ischemia. One of the possible drugs tested could be Methylprednisolone (MP). The results of our previous experiments showed that MP has the potential to reduce brain edema, but in these experiments we used purely experimental models of brain edema in rats – water intoxication model and osmotic BBB disruption model – without the occurrence of any pathological condition [4–8].

We venture to hypothesize that MP may have a similar effect in ischemic edema, given that its primary neuroprotective effect is attributed to its antioxidant capacity, which protects membrane lipids from peroxidation by free radicals, which are on the list of factors related to the development of ischemic edema [2,9]. In this experiment, we studied the effect of MP on ischemic brain edema after temporary occlusion of the middle cerebral artery for 90 min (MCAo90). We verified the presence of ischemic edema by determining the brain water content (BWC) by measuring dry/wet weight and by examining T2-weighted MR images, T2 relaxation times, and apparent diffusion coefficient (ADC). In another group, animals were administered MP 30 min after MCAo90, followed by 24 h reperfusion. Edema changes were documented by the same MRI examinations. The results of the experiment and the discussion dealing with the hypothetical effect of MP on ischemic brain edema are the subject of this paper.

## Methods

All experiments were approved by the Ethical Committee of the First Faculty of Medicine (Charles University in Prague) and were in agreement with the Guidelines of the Animal Protection Law of the Czech Republic and Guidelines for the treatment of laboratory animals EU Guidelines 86/609/EEC. For experiments, male rats of the Wistar strain weighing 400× g were used.

### Animals (MR scanning)

A total of 24 animals divided into three groups of eight animals in each group were used in the experiment. Specifically, these groups were: 1) control group in which animals were examined by MRI, referred to as CG in the text, 2) MCAo90m/24 h group in which animals underwent 90-minute MCA occlusion followed by 24-hour reperfusion. MRI examinations were performed on day 1 (after reperfusion) and subsequently on days 4 and 8. This group is referred to in the text as MCAo. 3) MCAo90m + 30 mMP/24 h group, in which animals were administered methylprednisolone 30 min after MCAo90 followed by 24 h reperfusion. MRI examinations were performed in the same schedule as in group 2). This group is referred to in the text as MCAoMP.

### Animals (Brain water content measurement – BWC)

Another 16 animals divided into two groups of eight animals in each group were used for the measurements. In the control group (CG) animals underwent BWC measurement and in the MCAo group animals underwent 90-minute MCA occlusion. The procedure for determining BWC consisted of the following steps – sacrificing the animal with a lethal dose of inhalation anesthetic – isoflurane 5 volume % (Forane ®, AbbVie Ltd.), decapitation, immediate removal of the brain, weighing the brain and determining the wet weight, placing the brain in a thermostat at 85 °C for six days, weighing the brain again and determining the dry weight, and determining BWC as a percentage according to equation: BWC (%) = (wet weight – dry weight)/wet weight × 100. According to the experimental protocol, the procedure was started after 24 h of reperfusion in the MCAo group [7,10].

### Insertion of the MCA occluder

Spontaneously breathing rat under the inhalation anesthetic isoflurane (Forane ®, AbbVie Ltd.) in concentration of 2 volume % underwent in the supine position the skin incision along the midline between the upper end of the sternum and the mandible. The left common carotid artery (CCA) and its branches (the internal carotid artery, ICA and the external carotid artery, ECA) were exposed with a standard microsurgical technique and ECA was ligated beyond the bifurcation. An intraluminal occluder (silicone rubber coated monofilament obtained commercially (www.doccol.com) was introduced into the CCA trunk from the arteriotomy and advanced until the origin of the middle cerebral artery (MCA) is blocked (distance from CCA bifurcation is 20–22 mm for a 400× g rat) and fixed with suture. The occluder was removed after 90 min, the distal and proximal ends of the exposed CCA were ligated, and the skin incision was closed with a continuous suture. After awakening, the animal was placed for 24 h in a home environment, at room temperature, with unlimited access to food and drink [11,12].

### Administration of methylprednisolone (MP)

In the MCAoMP group, MP (Solu-Medrol®, Pfizer) was administered at a dose of 100 mg/kg 30 min after MCAo90 *via* the intraperitoneal route. Being a steroid, MP has a lipophilic nature and is only weakly soluble in water. For being distributed in the body fluids it has to be in the form of ester methylprednisolone sodium succinate (MPSS). MPSS is not stable and due to activity of hepatic esterases, MP is released and subsequently bound to plasma proteins in the ratio of 40 to 60 %. Contrary to the free liposoluble MP, the high-molecular complexes (MP = molecular weight >50 kDa) cannot cross the blood-brain barrier. Only about one half of the total MPSS administered intravenously or intraperitoneally can cross the BBB. MP is metabolized in the liver with a mean elimination half-life in the range from 1.8 to 5.2 h.

https://go.drugbank.com/drugs/DB00959 [13]

https://labeling.pfizer.com/ShowLabeling.aspx?id=15288 [14]

MP was administered intraperitoneally 120 min after induction of ischemia, a time when BBB permeability is demonstrably increased, allowing high molecular weight substances to enter the brain [12,15,16].

### MR scanning

MR imaging was performed using a 7 T MR scanner MRS*DRYMAG 7.0T (MR Solutions, Guildford, United Kingdom) equipped with a rat head resonator coil.

The imaging protocol included basic T2-weighted turbo-spin echo sequence in sagittal and coronal directions (effective echo time *TE*_eff_=45 ms, repetition time *TR*=4000 ms, turbo factor *TF*=8, number of acquisitions *NA*=1, 13 slices, slice thickness 1 mm, field of view *FOV*=35×70 mm^2^ – coronal, *FOV*=37.5×60 mm^2^ – sagittal, matrix 128×256), axial T2-weighted turbo-spin echo sequence with higher resolution (*TE*_eff_=45 ms, *TR*=4500 ms, *TF*=8, 33 slices, slice thickness 0.5 mm, *FOV*=40×40 mm^2^, matrix 256×256), CPMG sequence for T2-mapping (echo spacing *TE*=9 ms, *TR*=4200 ms, 7 slices, slice thickness 1 mm, *FOV*=40×40 mm^2^, matrix 128×128, *NA*=1) and diffusion-weighted sequences (echo planar imaging sequence, *TE*=27 ms, *TR*=3000 ms, *NA*=2, diffusion gradient in one direction, seven *b*-values: 0, 100, 200, 400, 800, 1600, 3200 s/mm^2^, 9 slices, slice thickness 1 mm, *FOV*=35×35 mm^2^, matrix 100×94) for evaluation of ADC maps.

T2 and ADC maps were calculated using an in-house VIDI program [17] written in Matlab (MathWorks, Natick, MA, USA). Relaxation times and diffusion coefficients were evaluated in axial slices in the middle between the lambda and bregma in both hemispheres.

### Anesthesia

Animals were anesthetized by spontaneous inhalation of isoflurane (Forane®, AbbVie Ltd.) in air (3 % for induction, 1.5–2 % for maintenance) during invasive procedures – insertion of the MCA occluder, and MR examinations.

### Statistical analysis

Differences were analyzed using an unpaired two-tailed *t*-test. p<0.05 was considered as statistically significant.

## Results

**Fig. 1:**
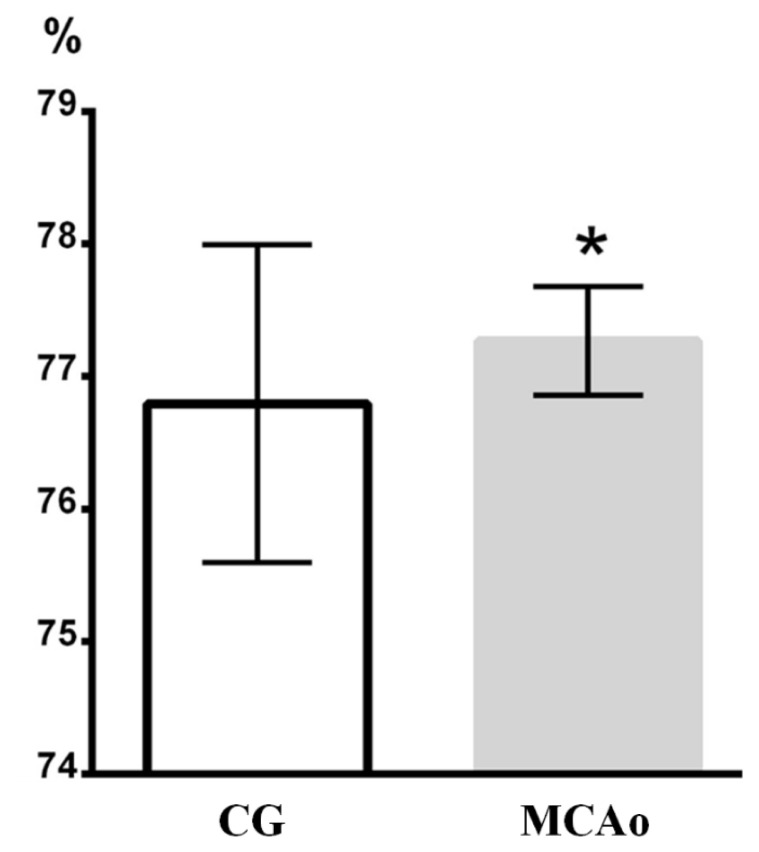
Brain water content – BWC (%). x-axis: BWC (%), y-axis: columns with average value ± SD, CG=control group (intact animals); MCAo=MCAo90 m/24 h group in which animals underwent 90-minute MCA occlusion followed by 24-hour reperfusion. (*) denotes statistically significant difference (p<0.05).

The results of the Brain water content measurement measurements are shown in Figure 1.

A statistically significant increase in Brain water content, found between the group of animals with induced ischemia (MCAo) and intact animals (CG), demonstrated the presence of ischemic edema in the first group.

**Fig. 2:**
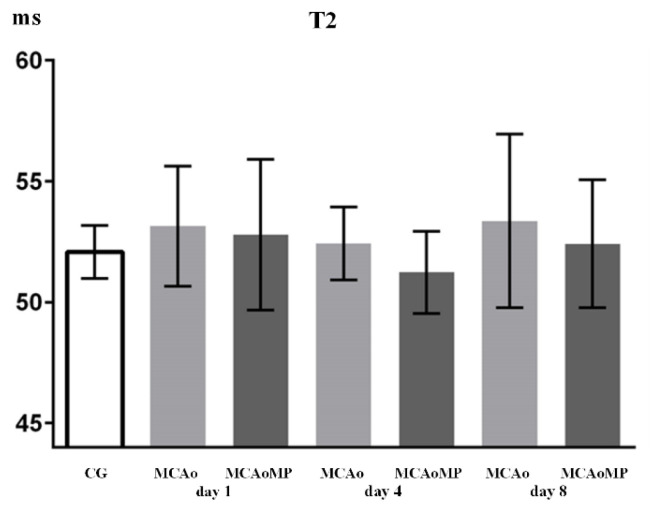
x-axis: T2 (ms), y-axis: columns with average value ± SD, CG = control group (intact animals); MCAo = MCAo90 m/24 h group in which animals underwent 90-minute MCA occlusion followed by 24-hour reperfusion; MCAoMP = MCAo90 m + 30 m MP/24 h group, in which animals were administered MP 30 min after MCAo90 followed by 24 h reperfusion.

**Fig. 3:**
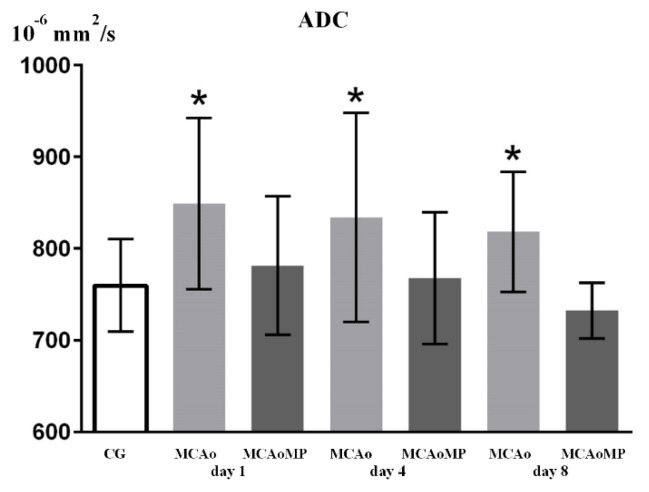
x-axis: ADC (10^−6^ mm^2^/s); y-axis: columns with average value ± SD, CG=control group (intact animals); MCAo=MCAo90 m/24 h group in which animals underwent 90-minute MCA occlusion followed by 24-hour reperfusion; MCAoMP = MCAo 90 m + 30 m MP/24 h group, in which animals were administered MP 30 min after MCAo90 followed by 24 h reperfusion. (*) denotes statistically significant difference (p<0.05).

Changes in T2 and ADC values in all groups are summarized in Figure 2 and Figure 3.

An edema was visible on T2-weighted images post MCAO in several animals only from both treated and untreated groups. Interestingly, in different animals, the edema evolved in different brain areas. The edema spanned through both hemispheres. However, quantification of T2 and ADC revealed subtle changes undetectable by standard T2-weighted images, but – due to sporadically observed edema, the average data had high variance.

An increase in the T2 value was observed on all measurement days in the MCAo group, which may indicate the presence of vasogenic edema, but a statistically significant difference between the MCAo and MCAoMP groups is not demonstrated due to the large variance of the measured T2 values in individual animals.

An increase in the ADC value is evident all measurement days in the MCAo group, indicating the presence of vasogenic edema. A statistically significant difference is demonstrated between the MCAo and MCAoMP groups, but there is no statistically significant difference between the CG and MCAoMP groups. This indicates a reduction in edema due to MP.

## Discussion

In general, the pathological mechanisms of ischemic edema represent today’s concept of brain edema after loss of energy supply. Ischemic edema is divided into three groups according to the molecular pathophysiology: cytotoxic edema, ionic edema, and vasogenic edema. Cytotoxic edema occurs rapidly after stroke, and is followed by ionic edema, vasogenic edema, and then mixed edema. While the first two types of edema develop within minutes of an ischemic stroke, the other two require several hours [18].

The blood-brain barrier (BBB) plays a key role in ischemic edema, especially towards an increase in brain water content and the possible development of life-threatening intracranial hypertension. Cerebral ischemia results in leakage of the BBB. Chemicals, liquids, and blood-borne cells enter the brain parenchyma through the damaged BBB, change the water and ion homeostasis in the brain, resulting in brain edema [3].

The mechanisms of ischemic edema are very complex and many factors are related to its formation [2]. We list only the most frequently mentioned factors that contribute to the development of ischemic edema. AQP4 (aquaporin-4), which is expressed by astrocytes, plays a bidirectional role in water transport and participates in the formation and elimination of brain edema. The significantly high expression of AQP4 after ischemic stroke may promote the formation of ischemic edema [19]. The SUR1-TRPM4 (Sulfonylurea receptor 1-transient receptor potential melastatin 4) channel contributes to the formation of ionic edema by regulating the Na^+^ inflow over the luminal membrane and the Na^+^ outflow over the abluminal membrane. SUR1-TRPM4 expression is upregulated in ischemic stroke [20].

MMPs (Matrix metalloproteinases) can mediate the destruction of basement membrane proteins, leading to increased permeability of the BBB, exudation of leukocytes, cerebral edema, and hemorrhagic transformation. MMP expression levels are very low under normal conditions, but the levels of MMP2 and MMP9 increase significantly within hours of cerebral ischemia [21]. PICs (peripheral immune cells) including neutrophils, monocytes, and T lymphocytes are activated by ischemic insult, lead to secretion of inflammatory molecules, thereby increasing BBB permeability [22]. CICs (cerebral immune cells) including microglia, astrocytes, and pericytes of the BBB, play a profound immunomodulatory role in ischemic stroke. Microglia and astrocytes are activated within minutes after cerebral ischemia and release some pro-inflammatory factors, such as TNF-α, NF-κB, IL-1β, and IL-6. They promote the inflammatory response, destruction of the BBB structure, increase of the BBB permeability and ischemic edema [23]. Cerebral ischemia/reperfusion injury occupies a special position among the factors causing ischemic edema. It is caused by a series of pathological cascade reactions triggered by the recovery of oxygenated blood flow into the ischemic brain tissue, but at present, the relationship between reperfusion and cerebral edema is still not clear [24]. This is mainly due to the discrepancy between the findings of clinical and experimental studies. Clinical studies have not yet demonstrated a direct link between reperfusion and the development of ischemic edema [25]. In animal experiments, the transient MCAO model showed that there was significant cerebral edema after reperfusion, suggesting that reperfusion injury promoted the formation of cerebral edema [26]. This may be related to the oxidative/nitrosative stress reaction after reperfusion, in which free radicals play an important role [27].

At present, osmotic diuretics, particularly mannitol and hypertonic saline, remain the main drugs used clinically to reduce ischemic brain edema. The main mechanism involves establishment of an intravascular osmotic gradient, resulting in water movement from the intercellular space to the intravascular space. On the other hand, it is currently a research hotspot for selecting targets and studying new drugs to prevent and treat cerebral edema based on the underlying molecular mechanisms. These include various inhibitors and blockers of the above-mentioned factors, but also drugs directed against free radicals, namely edaravone, uric acid, and citicoline [2,27]. The aforementioned discrepancy between the relationship of reperfusion and ischemic edema in clinical settings and in experimental models is probably the reason why corticosteroids are not used in the treatment of ischemic stroke in humans [28,29].

On the other hand, it is precisely MP whose effect was once verified in a randomized trial for the treatment of spinal cord injury [30]. Although this effect was later questioned, it became the inspiration to start testing the possibilities of MP in our experimental model of cerebral edema.

In our laboratory, we have used two experimental models to study brain edema in rats that induce the development of edema in a different way – the water intoxication (WI) model and the BBB osmotic disruption (BBBd) model [6]. WI induces hyponatremia, which controls the influx of Na^+^ and water into cells. The modified method of WI is based on intraperitoneal (i.p.) administration of distilled water in the total amount corresponding to 20 % of body weight in three consecutive doses over 24 h with a simultaneous administration of desmopressin. Each sub-dose represented one-third of the total dose of 0.032 mg/kg of desmopressin (1-desamino-8-D-arginine vasopressin) (OCTOSTIM®, Ferring). Desmopressin is an antidiuretic hormone, which potentiates the effect of hyperhydration by inducing hyponatremia [31]. The BBBd model is based on the intracarotid injection of a hyperosmolar solution, which leads to endothelial cell shrinkage and a temporary increase in BBB permeability. An intraluminal catheter is introduced into the ICA trunk through the arteriotomy for injection of Mannitol 20 % (200 g in 1000 ml of water for injection, 1098 mosmol/l) in a dose of 5 ml/kg at a rate of 0.12 ml/s [32]. The presence of brain edema induced by both methods is demonstrable by increased brain water content (BWC), decreased CT density and intracellular distribution of high MW intravital tracers (e.g. Evans Blue EB) [6].

We then used both mentioned brain edema induction models to test the effect of MP in several other experiments. MP was applied very early – 30 s to 2 h – after the onset of edema at a sufficient dose of 50–100 mg/kg body weight A dose of 100 mg/kg was intended for intraperitoneal injection and a half dose was used for intraatrial injection into the internal carotid artery. We hypothesized that the amount of drug administered directly to the brain may be significantly less than that required to deliver the same drug to the systemic vasculature, and that drug administered into the carotid rapidly reached its high intracerebral concentrations. A reduction in brain edema was demonstrated in these experiments. The evidence was a decrease in the intracellular distribution of Evans blue in the brain [6], a decrease in brain water content [7], a decrease in intracranial pressure [4,8], and a decrease in brain edema on MRI [5]. From the results of these experiments, it is possible to assume that MP at least affects the permeability of the cytoplasmic membranes of brain cells. However, its primary effect on brain cells in a purely experimental model of brain edema without the presence of another pathological condition is still not fully elucidated.

In this experiment, we studied the effect of MP on ischemic brain edema after temporary occlusion of the middle cerebral artery for 90 min (MCAo90), which is a model of very common pathological condition in humans – ischemic stroke. The presence of edema after 24 h of reperfusion was confirmed by a higher Brain water content (Fig. 1).

Two measurements were used in MRI. Apparent diffusion coefficient (ADC) is an indicator of the magnitude of the diffusion of water molecules within the tissue, and diffusion imaging provides information about the cellular architecture such as cellular size, membranes and volume fraction. ADC increases with higher extracellular volume and amount of fluids, and it is reduced when cell swelling is observed due to narrowing of the extracellular space within the cerebral parenchyma. High ADC values correspond to vasogenic edema. T2 (transverse relaxation time of excited protons) is related to water content and vascular permeability and increased T2 values reflect the development of a vasogenic edema. Of the two measurements, the ADC is considered the more sensitive [33,34].

Significantly higher ADC values in the MCAo group (Fig. 3) thus demonstrate the presence of vasogenic edema. Statistically significant difference in ADC values between MCAo and MCAoMP groups and the same ADC values in the MCAoMP and CG groups show the ability of MP to reduce ischemic brain edema (Fig. 3). Slight increase in T2 values (Fig. 2) might indicate similar phenomenon, however, the difference was statistically insignificant due to the large variability of T2 values in individual animals in the compared groups.

It can be stated that the mentioned results document the reduction of ischemic brain edema when MP was used. In agreement with our results, there are other literature data that present a number of evidences for the neuroprotective effect of MP on the development of ischemic brain edema.

Bremer *et al.* studied ischemic cerebral edema in primates. Brain edema was induced in Macaca mulatta after regional cerebral ischemia produced by selective embolization of the internal carotid artery bifurcation. High dose steroids showed an ability to modify edema in the cortex, putamen, and white matter. However, animals treated with methylprednisolone rather than dexamethasone showed a better neurological recovery and smaller infarcts [35].

Katayama *et al.* tested MP in an experimental model of cerebral ischemia caused by bilateral common carotid occlusion in spontaneously hypertensive rats. MP significantly reduced water content and lactate concentration and maintained high ATP levels [36].

Kalayci *et al.* studied the efficacy of methylprednisolone, which is claimed to cause rapid congealing of membranes, and to protect the cells against the free radicals in an experimental model of hypoxic ischemic brain injury to the right hemisphere in 7-day-old rat pups produced by cauterization of the right common carotid artery followed by hypoxia in 8 % oxygen and 92 % nitrogen for 3 h. The water content in the right hemisphere was significantly lower in the methylprednisolone-treated pups [37]. de Courten-Myers *et al.* occluded the middle cerebral artery for 4 h in cats. Administering methylprednisolone at high doses early after onset of ischemia significantly reduces tissue injury in cats that survive 4 days of temporary middle cerebral artery occlusion [38]. Slivka and Murphy examined high-dose methylprednisolone treatment following permanent and temporary focal cerebral ischemia in the rat. High dose methylprednisolone decreased infarct volume following temporary, but not permanent, focal ischemia [39].

The main effect of MP on ischemic brain edema is attributed to its antioxidant capacity, which has been known since the 1980s and 1990s. The process of antioxidation protects cell membranes against damage mediated by free radicals released by unsaturated fatty acids oxidation going on in the cells in the presence of molecular oxygen. The main neuroprotective effect of MP rests in that it prevents irreversible lipid peroxidation and a cascade of events resulting from a shortage of sources of energy. This takes the form of a change in the flow of ions on the cell membrane leading to intracellular edema and accumulation of extracellular glutamate, which is released in considerable quantities from postsynaptic membranes. Activated glutamate receptors open calcium channels and set off calcium influx into the cell. High levels of intracellular calcium cause damage to the cytoskeleton beside activating phospholipase A. That, in turn, sets off a cascade of arachidonic acid metabolites while released toxic eicosanoids (prostaglandins, leucotriens and thromboxanes) induce aggregation of thrombocytes, vasoconstriction and intravascular thrombosis. In another effect, arachidonic acid metabolites release more lipid peroxides, thus completing membrane damage [40–45].

We can only assume that it is the antioxidant capacity of MP with its complex impact on cellular metabolism that affects the movement of water in the brain and reduces ischemic brain edema.

We consider our results in the context of literature data to be a contribution to the search for a suitable substance affecting the extent of ischemic brain edema independently of the focal cerebral ischemia itself.
